# BarkBase: Epigenomic Annotation of Canine Genomes

**DOI:** 10.3390/genes10060433

**Published:** 2019-06-07

**Authors:** Kate Megquier, Diane P. Genereux, Jessica Hekman, Ross Swofford, Jason Turner-Maier, Jeremy Johnson, Jacob Alonso, Xue Li, Kathleen Morrill, Lynne J. Anguish, Michele Koltookian, Brittney Logan, Claire R. Sharp, Lluis Ferrer, Kerstin Lindblad-Toh, Vicki N. Meyers-Wallen, Andrew Hoffman, Elinor K. Karlsson

**Affiliations:** 1 Vertebrate Genomics, Broad Institute of MIT and Harvard, Cambridge, MA 02142, USA; kmegq@broadinstitute.org (K.M.); genereux@broadinstitute.org (D.P.G.); jphekman@broadinstitute.org (J.H.); swofford@broadinstitute.org (R.S.); jturner@broadinstitute.org (J.T.-M.); jjohnson@broadinstitute.org (J.J.); jalonso@broadinstitute.org (J.A.); xue.li2@umassmed.edu (X.L.); Kathleen.Morrill@umassmed.edu (K.M.); perloski@broadinstitute.org (M.K.); kersli@broadinstitute.org (K.L.-T.); 2 Bioinformatics and Integrative Biology, University of Massachusetts Medical School, Worcester, MA 01655, USA; brittney.logan@umassmed.edu; 3 Baker Institute for Animal Health, College of Veterinary Medicine, Cornell University, Ithaca, NY 14853, USA; lja2@cornell.edu; 4 School of Veterinary and Life Sciences, College of Veterinary Medicine, Murdoch University, Perth, Murdoch, WA 6150, Australia; C.Sharp@murdoch.edu.au; 5 Departament de Medicina i Cirurgia Animals Veterinary School, Universitat Autonoma de Barcelona, 08193 Barcelona, Spain; Lluis.Ferrer@tufts.edu; 6 Science for Life Laboratory, Department of Medical Biochemistry & Microbiology, Uppsala University, 751 23 Uppsala, Sweden; 7 Baker Institute for Animal Health and Department of Biomedical Sciences, College of Veterinary Medicine, Cornell University, Ithaca, NY 14850, USA; meyerswallen@gmail.com; 8 School of Veterinary Medicine, University of Pennsylvania, Philadelphia, PA 19104, USA; hoffm018@upenn.edu; 9 Cummings School of Veterinary Medicine, Tufts University, Grafton, MA 01536, USA; 10 Program in Molecular Medicine, University of Massachusetts Medical School, Worcester, MA 01655, USA

**Keywords:** dog, expression, genome, annotation, ATAC-seq, RNA-seq, epigenomic, canine, comparative

## Abstract

Dogs are an unparalleled natural model for investigating the genetics of health and disease, particularly for complex diseases like cancer. Comprehensive genomic annotation of regulatory elements active in healthy canine tissues is crucial both for identifying candidate causal variants and for designing functional studies needed to translate genetic associations into disease insight. Currently, canine geneticists rely primarily on annotations of the human or mouse genome that have been remapped to dog, an approach that misses dog-specific features. Here, we describe BarkBase, a canine epigenomic resource available at barkbase.org. BarkBase hosts data for 27 adult tissue types, with biological replicates, and for one sample of up to five tissues sampled at each of four carefully staged embryonic time points. RNA sequencing is complemented with whole genome sequencing and with assay for transposase-accessible chromatin using sequencing (ATAC-seq), which identifies open chromatin regions. By including replicates, we can more confidently discern tissue-specific transcripts and assess differential gene expression between tissues and timepoints. By offering data in easy-to-use file formats, through a visual browser modeled on similar genomic resources for human, BarkBase introduces a powerful new resource to support comparative studies in dogs and humans.

## 1. Introduction

The domestic dog is an increasingly important model for a wide variety of human diseases, including cancer, immune-mediated disorders, and psychiatric diseases, as well as for healthy aging [1,2,3,4,5,6,7]. The pet dog population is unusual in that it is comprised in a large part of ancestry from dog breeds, which are genetically isolated populations that are just a few hundred years old and have limited genetic diversity [8,9]. Purebred dogs often suffer from a high risk of diseases that closely model complex human diseases, and the population structure of breeds can make genetic mapping approaches far more powerful [8,10]. With a very large population size (70 million in the United States alone), and a shared environment with human owners, dogs are, in many ways, an ideal model. However, as canine whole genome datasets expand [11,12], comprehensive epigenomic profiling in dogs lags behind, impeding the translation of genetic associations into functional understanding.

In human genomics, available large scale epigenomic resources such as ENCODE [13], GTEx [14], and the National Institute of Health (NIH) Roadmap [15] catalog the functional elements active in diverse, healthy tissues. Such resources have proven exceptionally powerful for investigating the non-coding regulatory variants that make up the vast majority of risk factors identified in complex-trait genomewide association studies (GWAS), for finding which candidate variants are most likely to perturb cell function, and for distinguishing which cell and tissue types are most likely to be involved in the disease process [16]. By integrating multiple different types of epigenomic data for each cell type or tissue, active, noncoding regulatory elements map far more specifically [17,18]. Developing similar resources for a natural model organism like a dog would support comparative studies of the genome regulatory function and the conservation of disease mechanisms, further elucidating human disease biology [19].

Currently, there is no canine equivalent to these human epigenomic resources. Canine genes and non-coding RNAs are mapped in an Ensembl gene annotation from July 2012 and a University of California Santa Cruz (UCSC) Genome Browser track hub from 2014, both based on the same canine RNA-seq dataset [20,21,22,23,24]. This dataset includes a range of diverse adult canine tissues (blood, brain, heart, kidney, liver, lung, ovary, skeletal muscle, skin, and testis). The Ensembl annotation includes 19,856 coding genes and 11,898 non-coding genes, and the UCSC track has 22,798 protein coding genes (20,657 of high confidence) and 7224 putative long noncoding RNAs [25,26].

The RNA-seq dataset used to build these existing annotations has several significant limitations. First, it includes only adult tissues, potentially missing critical genes or isoforms active only during development. Second, it contains just one sample for each tissue and so provides no biological replicates to validate the data, to examine how gene expression varies among individuals, or to determine which genes are consistently up or down regulated in individual tissue types. Finally, RNA-seq alone does not capture untranscribed regulatory elements.

For untranscribed regulatory elements, the canine research community relies on genome annotations mapped onto the dog genome from other species, including sequence conservation scores [27,28]. Yet, while most protein coding genes are expected to be conserved across mammals, certain types of regulatory elements, such as enhancers, turn over rapidly [29,30]. The lack of epigenomic data for dog tissues is particularly concerning as dogs are a natural model for complex, polygenic diseases associated primarily with regulatory, rather than coding, variation [7,11,16]. Even with the limited epigenomic resources available today, canine studies have yielded new insight into human diseases, including cancers [4,31,32,33,34,35,36,37], behavioral traits and disorders [2,38,39], autoimmune diseases [3,40], and others [41,42,43,44,45,46].

Here, we introduce BarkBase (http://www.BarkBase.org), a public resource that builds on the paradigm established by ENCODE and NIH Roadmap. At present, BarkBase contains RNA sequencing data from 27 diverse tissues collected from five adult dogs, paired with 30× whole genome sequence data. BarkBase also includes RNA sequencing data for embryonic tissues from five carefully staged gestational time points. We are currently generating and uploading assay for transposase-accessible chromatin using sequencing (ATAC-seq) data for each of the adult tissue samples, and anticipate that this will be completed by the end of 2019.

Our initial analyses of BarkBase data expand the catalog of dog genes, including noncoding genes, and identify novel regulatory elements from ATAC-seq peaks, confirming the value of BarkBase as a tool for canine genomics, and enhancing the value of the dog as a powerful natural model for the study of human disease.

## 2. Materials and Methods

### 2.1. Sample Collection

#### 2.1.1. Adult Tissue

**Figure 1:**
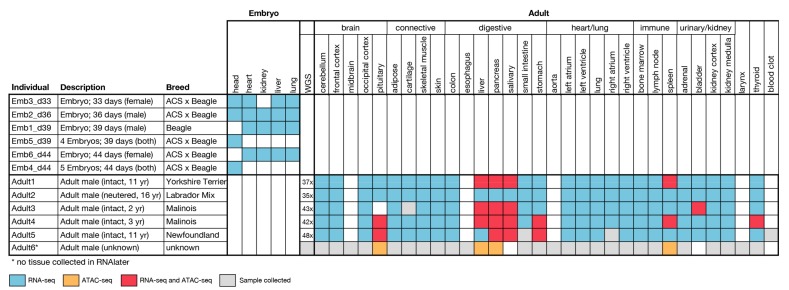
BarkBase sample collection and data production. Samples were collected from a total of six embryos, and six adult dogs. BarkBase currently contains RNA-seq data from up to five tissues in d33, d36, d39, and d44 embryos, and from up to 27 tissues sampled from each of five adult dogs diverse in age and in breed ancestry. ATAC-seq data are currently available for eight tissues from a subset of individuals. Additional data sets will be posted as they become available.

**Figure 2:**
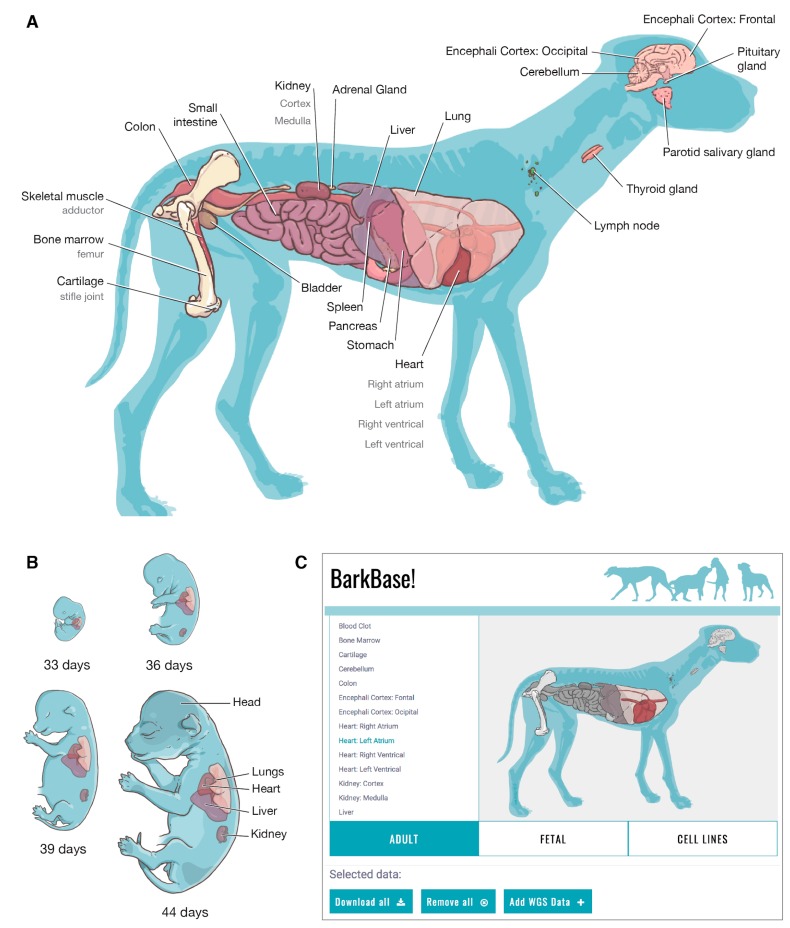
The BarkBase web portal. The BarkBase web portal enables download of whole genome sequence (WGS) data, RNA-seq data, and assay for transposase-accessible chromatin using sequencing (ATAC-seq) data for (**A**) up to 27 tissues from each of the five adult dogs; and (**B**) up to five tissues from canine embryos collected at each of the four staged gestational timepoints. Reads preprocessed and aligned to CanFam3.1 are available at BarkBase.org. From the BarkBase interface (**C**), users can readily select specific tissues and samples. Raw read data from RNA-seq and ATAC-seq is available through the Sequence Read Archive (SRA) (Table S1).

All research in this study was conducted according to NIH guidelines for the Care of Vertebrate Animals used in Testing, Research, and Training. Six dogs were enrolled through the Deceased Dog Donation program at Cummings School of Veterinary Medicine at Tufts University, with approval from the Institutional Animal Care and Use Committee (IACUC). All dogs were euthanized for medical reasons, and donated by owners after death. Inclusion was based primarily on availability, with the only exclusion criterion being a cancer diagnosis. By chance rather than study design, all dogs were male. They spanned a range of sizes, ages, and breeds, and included one mixed breed dog with a high proportion of Labrador Retriever ancestry (Figure 1, Table S1). We collected biopsies from up to 31 tissues (Figure 2), including heart (left atrium, left ventricle, right atrium, and right ventricle), brain (cerebellum, frontal cortex, occipital cortex, and pituitary), kidney (cortex and medulla), adipose tissue, adrenal gland, bladder, bone marrow, cartilage, colon, liver, lung, small intestine, lymph node, pancreas, salivary gland, skeletal muscle, skin, spleen, stomach, and thyroid gland. Sample collection was completed within two hours of death. Each sample was divided in two, with one half stored in RNAlater and the other flash frozen in liquid nitrogen, then stored at −80 °C until processed.

#### 2.1.2. Embryonic Tissue

All animal care and experimental protocols related to embryonic tissue collection were approved by the Institutional Animal Care and Use Committee at Cornell University (1989–0068). Embryos were collected using hysterotomy, separated through microdissection and individually stored in RNAse-free phosphate buffered saline (PBS, 4C), as described in Meyers-Wallen et al [47]. Each embryo was developmentally staged based on photographs of external morphology [48]. Sex was ascertained by PCR targeting the *SRY* locus. For two samples (heads), tissues from several individuals of the same litter were stored and processed together to ensure sufficient material (Figure 1).

### 2.2. Whole Genome Sequencing

#### 2.2.1. Sequencing and Variant Calling

Genomic DNA was prepared utilizing custom indices from Integrated DNA Techologies (Coralville, Iowa, USA) designed by the Broad Institute Genomics Platform (Cambridge, MA, USA) and a HyperPrep library construction kit from Kapa Biosciences (Wilmington, MA, USA). Sequenced was done on a NovaSeq 6000 (Illumina, San Diego, CA, USA), using two color chemistry at 2 × 150 bp reads to 30× coverage, de-multiplexed, aggregated, and aligned to the CanFam3.1 reference via BWA-MEM [49]. Read data was processed according to GATK best practices, specifically base quality score recalibration, variant identification using HaplotypeCaller in GVCF mode, and joint calling, using the GATK 6/24/2016 nightly build against a background of 872 additional dogs and other canids (Table S2) [50,51]. Variant filtration was accomplished by hard filtering. For SNPs, the filtration parameters were: QD < 2.0 or FS > 60.0 or MQ < 40.0 or MQRankSum < −12.5 or ReadPosRankSum < −8.0. For insertions and deletions, the filtration parameters were: QD < 2.0 or FS > 200.0 or InbreedingCoeff < −0.8 or SOR > 10.0 or ReadPosRankSum < −20.0.

#### 2.2.2. Relatedness

Relatedness of adult dogs was measured through pairwise identity by descent (IBD) estimation in Plink 2.0 [52] using the “genome” flag on 1,632,289 autosomal variants remaining after pruning variants in linkage disequilibrium. Pruning was done with the “indep” flag (window size of 50 variants, a window shift of five variants and variance inflation factor threshold of 2) [52,53].

#### 2.2.3. Annotation

Variants were annotated with 18 functional categories using SnpEff [54] and CanFam3 (v3.1.86) from Ensembl [22] as the reference database.

### 2.3. RNA Sequencing, Normalization, and Analysis

#### 2.3.1. Sequencing

RNA samples with an RNA quality score (RQS) >5.5 were poly-A selected, strand-specific cDNA synthesized, and an Illumina library constructed via the Illumina TruSeq protocol. Libraries were size selected (450–550 bp inserts) and sequenced on an Illumina HS2500 (2 × 101 bp reads) to >50 million reads/library. We achieved a median of 64 million reads, substantially exceeding the ENCODE standard of 30 million reads per sample [55].

#### 2.3.2. Read Preprocessing

RNA-seq reads were analyzed by the workflow outlined by Pertea et al. [56]. Briefly, reads were aligned to the CanFam3.1 dog genome using HISAT2 [56,57] in dta mode. Transcripts were assembled from the aligned reads using StringTie [56,58], with a gene annotation from Ensembl as a reference (version 95). Transcripts from all samples were merged using StringTie to create a new reference file, which was then used to estimate transcript abundance in each sample. Raw read counts at both the gene and transcript level were generated from StringTie transcript abundances using the “prepDE.py” Python script provided in the StringTie online manual (https://ccb.jhu.edu/software/stringtie/dl/prepDE.py), using the −l flag to set read length to 101 base pairs. The raw read counts at the gene and transcript level are available at https://data.broadinstitute.org/barkbase/.

#### 2.3.3. TMM Normalization to Calculate Counts Per Million (CPM)

The trimmed mean of M (TMM) normalization method [59] as implemented in the R package edgeR [60,61] was used to normalize raw RNA-seq read counts. TMM normalization helps avert underestimation of the abundance of lowly or moderately expressed genes in samples with very high expression from a subset of genes, thereby avoiding inflation of the number of genes inferred to be differentially expressed between samples. A TMM normalization factor was calculated for each sample, then applied to calculate counts per million (CPM) from raw read count. For most analyses, transcripts were kept if they were expressed at >0.16 counts per million (CPM) in two or more samples, which is equivalent to requiring approximately 10 reads in our median library size of 64 million [62]. For the comparison with human RNA-seq data, a more stringent cutoff of 1 CPM in two or more samples was implemented. Filtered, normalized CPM counts at both cutoffs are available at: https://data.broadinstitute.org/barkbase/.

#### 2.3.4. Cumulative Abundance and Tissue-Specific Reads

CPM values were used to calculate overall fraction of the transcriptome contributed by each gene. Genes were sorted by CPM, the cumulative sum calculated, and the fractional contributions of the 1000 top-contributing genes plotted for each sample.

#### 2.3.5. Hierarchical Clustering of Samples and Adult Dogs

Euclidean distances among CPM values for the samples from adult dogs and from embryos were calculated using the dist function in R. Overall distances among the adult dogs were calculated by concatenating CPM values for the 23 tissues for which data were available from all five individuals, and distances calculated as for the single-tissue samples. Hierarchical clustering was performed using the hclust function in R [63].

#### 2.3.6. Differential Gene Expression (DGE) and Enrichment Analysis of Embryonic Data

The edgeR package [60,61] was used to identify genes differentially expressed between embryonic time points d36 and d44 in head, liver, lung, heart, and kidney. At present, we have access to only one carefully staged embryonic sample for each time point. To accommodate the lack of biological replicates, we instead calculated dispersion values using data from 3027 genes from among the 3119 that were previously identified as "housekeeping genes" in human RNA-seq data [64] and that were also expressed in dog. Significance scores calculated in edgeR and in parallel using a different inference package in R, DESeq2 [65], were strongly correlated (*p* < 2.2 × 10^−16^). We defined genes with false discovery rate (FDR) < 0.1 in the edgeR-adjusted results as differentially expressed between d36 and d44. Ingenuity Pathway Analysis (IPA) from Qiagen (Hilden, Germany) was used to identify enrichment of specific categories within "Diseases or Functions Annotation”.

#### 2.3.7. Comparison of Gene Expression between Dog and Human Tissues

Human RNA-seq data from 53 tissues of 714 human donors was downloaded from the GTEx Portal (GTEx Analysis V7, https://gtexportal.org/home/datasets). The raw human read counts from GTEx were converted to CPM using the TMM normalization method described above. Samples within a species were grouped by tissue type and the expression summarized using the median CPM for each gene. The Ensembl reference annotation was previously provided to StringTie as part of the transcript merging process, during which StringTie annotated transcripts with matching Ensembl gene names. These gene names, as well as cross-species orthology data downloaded from Ensembl [66], were used to map between BarkBase genes and human genes in GTEx. All BarkBase genes not uniquely annotated with an Ensembl gene name were discarded, as were all genes that did not uniquely map between human and dog. For each tissue, genes with median CPM ≤ 1 in both species were discarded. The Spearman correlation between the two species was then calculated using scipy [67]. The correlation matrix comparing dog and human tissues includes all tissue types for which we had data in both species.

#### 2.3.8. Comparison of Transcript Set to the Existing Ensembl Canine Reference Annotation

The GffCompare tool [68] was used to compare the transcripts annotated by StringTie to the Ensembl canine reference annotation. Transcripts were annotated as overlapping if they completely or partially overlapped a reference transcript (GffCompare class codes “=”, “c”, “k”, “m”, and “j”), excluding cases where the overlap was on the opposite strand, completely contained within an intron of the reference transcript, and in cases where the reference was completely contained within an intron of the StringTie transcript. A reference track containing non-dog RefSeq genes aligned to the canine reference (“Other RefSeq” track) was downloaded from the UCSC Genome Browser [24,69,70,71] in General Transfer Format (GTF) using the table browser. Human genes in this track were annotated using Ensembl BioMart [66]. Novel transcripts were identified as those not overlapping either annotation (class code “u” in the GffCompare output). Enrichment analysis was performed using the GOseq [72] Bioconductor [73] package. Canine genes were first mapped to 1:1 human orthologs using Ensembl BioMart, and the human gene names input into GoSeq. Enrichment was tested using the set of all canine Ensembl genes that map to 1:1 human orthologs as the background. Using this approach, multiple canine snoRNAs mapping to a single human gene name were collapsed for the enrichment analysis. The Wallenius approximation was used to correct for gene length. Multiple testing correction was performed using the Benjamini–Hochberg procedure, with significance set at an FDR of 0.05. The genomic coordinates covered by BarkBase and the Ensembl and Hoeppner et al. annotations were compared using the BEDTools [74] intersect tool using the −s flag for strand specific comparisons. The GTF files annotating each dataset were first converted to BED files. The BEDTools merge tools was used to create unique non-overlapping intervals within each file. Each merged BED file was then compared to each of the others using the BEDTools intersect tool, and the number of base pairs covered by the intersecting intervals summed.

#### 2.3.9. Comparison of Unannotated Transcripts to the RefSeq Vertebrate Mammalian Proteins

Multi-exonic transcripts not matching either the Ensembl or Hoeppner et al. annotation were aligned using blastx 2.2.30 [75] to the RefSeq vertebrate mammalian protein database, release 93 [76]. Query transcripts were considered to significantly match a RefSeq protein if blastx returned a match with a bit score greater than 60.

### 2.4. ATAC-seq

#### 2.4.1. Sample Preparation and Sequencing

To extract cellular nuclei, each tissue sample was homogenized in an EZ lysis buffer using Kimble Dounce All-Glass Tissue Grinders, starting with a small (pea-sized) piece of tissue. The sample was incubated on ice for five minutes, poured through a cell strainer, and then pelleted and resuspended in 150 μL of resuspension buffer (Nuclei Isolation Kit: Nuclei EZ Prep from Sigma-Aldrich, St. Louis, MO, USA). To determine the volume needed for 50,000 cells, 10 μL of resuspension buffer was mixed with 1 μL of Trypan Blue and cells counted on a hemocytometer. The needed volume of the cell preparation was centrifuged, the resuspension buffer removed, and 50 μL of the master mix (25 μL 2x TD buffer, 5 μL Tn5, and 20 μL distilled H_2_O) added. The sample was mixed by vigorous pipetting and incubated at 37°C for 30 min while rocking. The Qiagen MinElute Reaction Cleanup Kit was used to elute the pure DNA into 10 μL of elution buffer. The sample was then transferred to a 96-well plate and amplified via PCR to create the final libraries. The libraries were quantified using the BioAnalyzer 2100 from Agilent Technologies (Santa Clara, CA, USA) and the Kapa Library QuantKit, and sequencing was performed on two lanes of the HS2500 Rapid Run 2 × 25. We averaged around 80 million autosomal reads per sample, exceeding the ENCODE minimum recommendation of 50 million reads [55].

#### 2.4.2. Processing Reads, Calling Peaks, and QC of Libraries

Reads were processed and peaks called following the ATAC-seq guidelines developed by John M. Gaspar [77]. Briefly, reads were aligned to CanFam3.1 using Bowtie2 in “very sensitive” mode. Mitochondrial reads were then removed using Samtools. PCR duplicates were removed using Picard tools, and non-uniquely mapping reads were removed using Samtools. Finally, bam files were converted to bed coverage interval files using the SAMtoBED.py script by John M. Gaspar. Quality metrics including peak counts, transcription start sites (TSS) scores, and mapping stats were derived using ATAQV [78]. As the existing gene annotation for dog is not as comprehensive as the human annotations, we could not directly apply the transcription start site enrichment score thresholds recommended by ENCODE [55]. Instead, we removed all samples falling below one standard deviation less than the mean, which left us with 37 samples with TSS scores of 1.3 or higher (mean of 2).

#### 2.4.3. Assessing Overlap of Peaks

The BEDTools intersect tool [74] was used to compare overlaps of called peaks among and across tissues. Subsequent intervals were annotated and analyzed using the ChIPseeker R package [79], in conjunction with annotations from Ensembl [21,80]. This tool retrieves the nearest genes around an ATAC-seq peak.

#### 2.4.4. Data Sharing

Data files are available for download at our ftp site (https://data.broadinstitute.org/barkbase) and analysis scripts at https://github.com/broadinstitute/barkbase_paper_analysis/.

## 3. Results

### 3.1. BarkBase Website

We collected samples of 33 adult tissue types, with 28 tissues sampled from at least five individuals ( 1,2 A). We also collected five tissues (head, heart, kidney, liver, and lung) across four embryonic time points ( 1,2 B). To date, we have completed RNA sequencing of 150 samples (27 adult tissues and five embryonic tissues) and have generated ATAC-seq data for 36 samples (15 adult tissues); 22 adult samples (nine tissues) currently have both data types. The embryonic dataset, which includes only RNA-seq data, is complete, with data for all tissues and timepoints except for one kidney sample, which failed to yield RNA of adequate quality for sequencing. Data generation (RNA-seq, ATAC-seq, and whole genome sequencing (WGS)) in the adult samples is ongoing.

To share this data, we developed a website inspired by the data access portal for the NIH Roadmap project [81] (Figure 2C). Like the Roadmap Visual Browser, BarkBase includes anatomical illustrations of the samples, both at each embryonic time point and in the adult dog, displaying the scope of the data set. By selecting specific tissues and samples, users can construct a customized dataset for download. BarkBase.org provides GTF formatted files for RNA-seq and ATAC-seq, and Variant Call Format (VCF) files for WGS. The total size of the data available through BarkBase.org is currently ~7 gigabytes, with more data being generated. The capacity to subset data prior to download can substantially reduce download times. The read data, aligned to CanFam3.1, is available through the Sequence Read Archive [82].

### 3.2. Whole Genome Sequencing

For each of the five adult dogs with nearly complete RNA-sequencing data, we have also generated high coverage whole genome sequencing of the germline DNA. For each dog, we sequenced DNA extracted from cerebellum tissue to 35–48× coverage (average coverage 41×). Across these five samples, we successfully called 56,244,173 of 57,498,383 possible variant sites (call rate = 97.82%), of which 4.74% were heterozygous and 3.1% were homozygous non-reference. Annotation of the 8,147,173 sites with non-reference allele frequency >0, using SnpEff [54], classified 53.5% (4,352,137) as intergenic, 32.25% (2,624,663) as intronic, 12.63% (1,028,340) as near genes, and 1.74% (142,033) as being either in transcribed regions or disrupting splicing.

### 3.3. Overlap With Existing Gene Annotations

There are two existing gene annotations for CanFam 3.1. The most widely used is an Ensembl annotation generated using a standard Ensembl mammalian genebuild pipeline. It incorporated RNA-seq data, provided by the Broad Institute, for one sample of each of ten tissues (blood, brain, heart, kidney, liver, lung, ovary, skeletal muscle, skin, and testis) [21]. RNA-seq libraries for this dataset were generated using two RNA selection techniques (poly-A and duplex-specific nuclease), adding sensitivity particularly for shorter noncoding transcripts. The Ensembl gene build contains 39,074 transcripts in 32,704 genes (60 Mb total), including 19,856 coding, 11,898 non-coding, and 950 pseudogenes. This same RNA-seq data was also used for the Hoeppner et al. improved canine genome annotation, published in 2014, which used a less conservative 194,671 transcripts in 22,172 coding genes, 7224 lincRNA candidates, and 5295 antisense transcripts, as well as 82,039 other transcripts (249 Mb total) [20].

**Figure 3:**
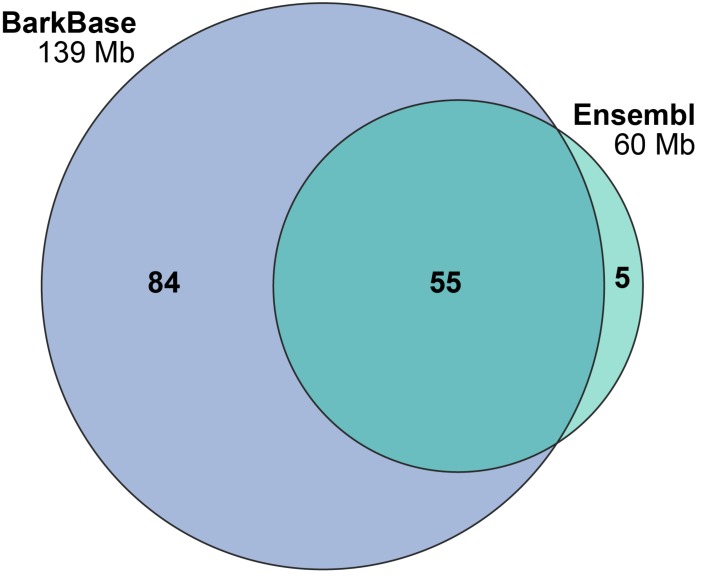
BarkBase captures novel transcripts. Overlapping the transcriptome from BarkBase and Ensembl shows most bases are captured in both datasets. BarkBase contains 84 Mb of transcribed sequence not included in the existing annotation, highlighting its utility to improve the annotation of the canine genome.

Our new BarkBase RNA-seq data, generated from libraries made using poly-A selection only, contains 151,787 transcripts in 37,106 StringTie-assembled genes. On the single base level, there is a high degree of overlap (over 90%) with the Ensembl canine reference annotation. In addition, BarkBase contains 84 Mb of unique sequence, compared to 5 Mb in the Ensembl annotation. (Figure 3). BarkBase also has a large degree of overlap with the Hoeppner et al. data set (103 Mb), with the Hoeppner et al. data containing an additional 146 Mb of sequence.

We examined the sensitivity of BarkBase, the Ensembl annotation, and the Hoeppner et al. data to identify exons present in the other data sets (Table S3). Sensitivity was calculated as the total number of exons in the reference annotation identified by the query dataset, divided by the total number of exons in the reference dataset [68]. BarkBase captures most of the Ensembl exons (87.6%) and about half (56.6%) of the exons in the Hoeppner et al. annotation. Reciprocally, both the Ensembl and Hoeppner et al. annotations captured about half of the exons in BarkBase (47.1% and 55.2%, respectively). This suggests that BarkBase includes exons missed in previous annotations, and will expand the catalog of annotated dog exons, transcripts, and genes.

Of the 32,704 Ensembl dog genes (19,856 coding, 11,898 non-coding, and 950 pseudogenes), 69% overlapped at least one transcript in BarkBase (Table S4). We capture the large majority of coding genes annotated in Ensembl (90%), but only 34% of the non-coding genes. The genes missing from BarkBase are largely non-coding genes without human orthologs. Of the 10,118 Ensembl genes that are not in BarkBase, 77% are noncoding, compared to only 18% of those that are in BarkBase. The genes missing from BarkBase are also less likely to have a 1:1 human ortholog (13% vs 67%).

There are 1314 Ensembl dog genes (44% coding) that have human orthologs but are missing from BarkBase (Table S5). We performed enrichment analysis of this gene set using GOseq and found 52 significantly enriched Gene Ontology (GO) terms. The top scoring term was “olfactory receptor activity” (*p* = 1.8 × 10^−104^, Table S6), followed by other terms relating to olfaction and sensory processes, including “detection of chemical stimulus involved in sensory perception of smell” (*p* = 1.8 × 10^−104^) and “G-protein coupled receptor activity” (*p* = 1.4 × 10^−63^). Less enriched terms include “mRNA binding involved in posttranscriptional gene silencing” (*p* = 2.4 × 10^−12^), “sexual reproduction” (*p* = 5.0 × 10^−3^) and “fertilization” (*p* = 6.5 × 10^−3^), and “male gamete generation” (*p* = 4.6 × 10^−2^). This suggests that many of the Ensembl genes not seen in BarkBase may be missed due to the lack of testis tissue in BarkBase, as testis is known to express olfactory [83] as well as taste receptors [84].

In contrast, the Ensembl genes found in BarkBase are highly enriched for GO terms related to broadly important cellular processes, including “catalytic activity”, “protein binding”, “intracellular”, “cell”, “nucleus”, and “cytoplasm.” All six terms had a reported *p*-value of 0 (Table S7). This enrichment is likely due to the gene set being tested contains 69% of all genes in the Ensembl reference, particularly coding genes. Many gene sets in the GO terms have all or nearly all members represented in this set, leading to highly significant enrichment of larger gene sets.

BarkBase captures 14,518 transcripts in 10,691 genes that are missing from the Ensembl canine annotation. Of these, 8051 are multi-exon transcripts. Approximately 80% of these were captured in Hoeppner et al., but we found 1520 novel transcripts. Of these, most (1090) are also missing from another recently published catalog of canine long non-coding RNAs [25]. Using BLAST, we tested all our genes against known protein-coding sequences, finding 769 potential novel lncRNAs (GTF file available at https://data.broadinstitute.org/barkbase/).

Of the transcripts that did not overlap the Ensembl or Hoeppner annotations, 15 transcripts overlapped five known human protein coding genes: The salivary protein encoding gene *STATH*, the eosinophil expressed pathogen response gene *EPX*, homeobox transcription factor *HOXD13*, transcriptional repressor *FEZF1*, and the calcium signaling gene *AHNAK2*. Of these human genes, four are not annotated in the canine genome, while *AHNAK2* does not map uniquely, perhaps indicating a set of paralogous sequences in the dog. *AHNAK2* is annotated at two locations, and our data adds two 5’ exons to the current gene annotation (Table S8).

### 3.4. Variability Among Adult Dogs and Tissues

BarkBase includes data for up to five dogs per tissue, offering opportunity to assess how patterns of expression vary between individuals and between healthy tissues. Previous datasets, were either disease-focused (primarily cancer-focused) or included just one individual per tissue [20,32,85,86,87,88]. With our current data set, we have sufficient power to detect over 80% of genes with at least a two-fold change in expression between tissues, but are still underpowered to comprehensively detect differentially expressed genes [89]. We can also assess a correlation of gene expression patterns between tissues on a whole transcriptome level.

**Figure 4:**
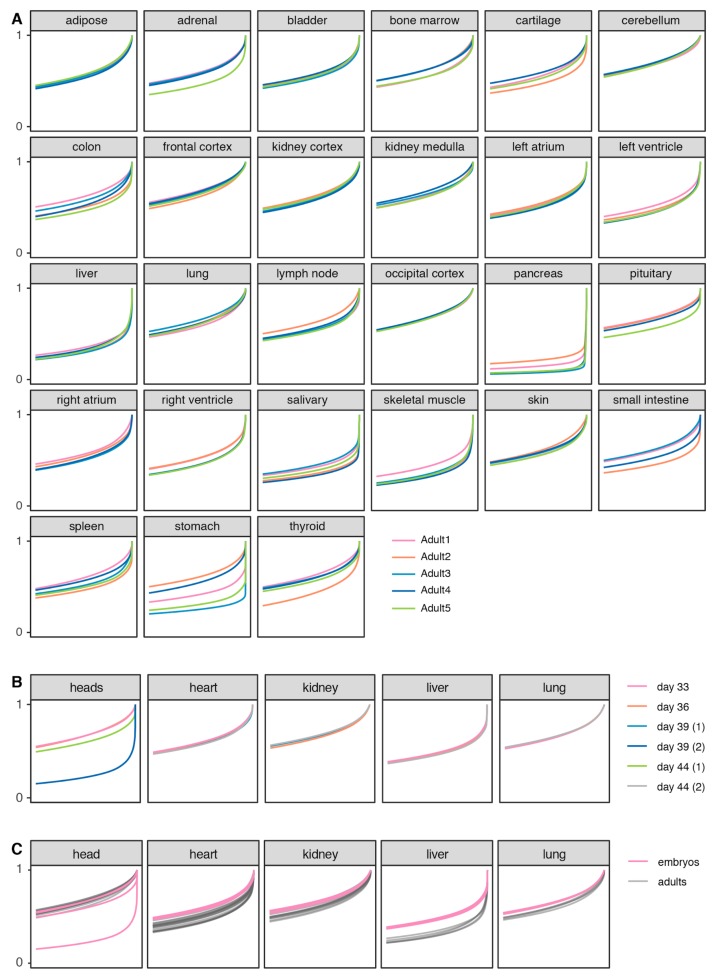
Cumulative transcriptome expression is matched to tissue type. Cumulative sum of fraction of tissue-specific transcriptomes represented by individual genes in (**A**) canine embryos at four gestational time points; and (**B**) up to five individual adult dogs. Single-gene counts per million (CPM) values were divided by sample-sum CPM, sorted in increasing order, and the cumulative sum calculated. Cumulative values are shown for the 1000 top-expressed genes in each sample. Data sampled from a given embryonic tissue at different gestational time points are very similar, perhaps reflecting the fairly narrow time window of sampling. Combining data from adult and embryonic samples (**C**) reveals strong similarity of data from given tissue types across individuals and developmental stages.

Overall, we found that in adult dogs, gene expression is tightly matched to tissue type. We first looked at the fraction of the transcriptome contributed by the most highly expressed genes, an approach used to assess similarity in human tissue samples [90]. We saw clear similarity among dogs and diversity between tissues. In all of the tissue types, the transcriptome is dominated by a fairly small number of genes, with the top 1000 expressed genes comprising at least 25% of transcripts in all but one of the tissues types (Figure 4A). In pancreas, the pattern is even more extreme, with 75% of transcripts coming from just 40 genes, broadly consistent with findings from the human pancreas [91]. In the embryonic tissues, the pattern of strong similarity within tissues persisted, even though the samples were from different embryonic timepoints (Figure 4B). This may reflect the fairly narrow window of gestational ages sampled, or that normal embryonic development involves changes affecting just a small subset of transcribed genes. The embryonic tissues were also notably similar to their adult counterparts, suggesting the similarity among embryonic timepoints reflects their having acquired transcriptomic features of the relevant adult tissue type (Figure 4C). A single embryonic head sample is an outlier, with the top 1000 genes contributing a lower proportion of expressed transcripts, a pattern that could result if a less homogenous tissue sample were obtained during microdissection.

**Figure 5:**
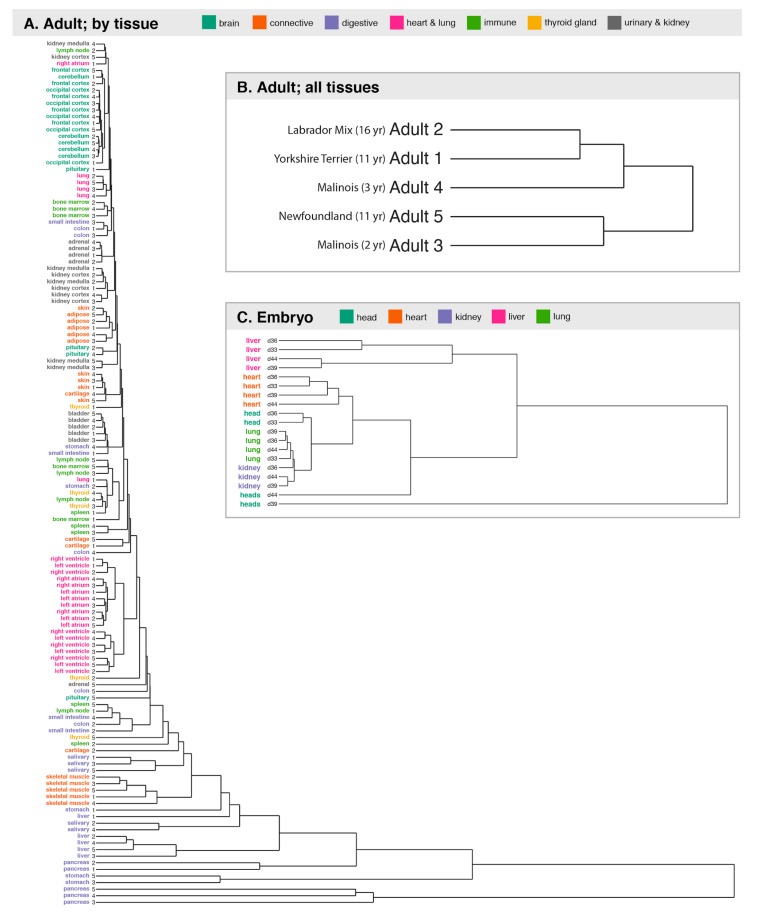
Transcriptome data from five individuals clusters primarily by tissue type. Hierarchical clustering of RNA-seq data from (**A**) single tissues of five adult dogs; (**B**) five adult dogs, based on data concatenated across 21 tissues; and (**C**) embryonic tissues sampled at four gestational time points. Clustering is based on Euclidean distances among samples. Overall, in data from both adults and embryos, samples of a given tissue cluster across individuals. As observed in cumulative analysis, embryonic samples of a given tissue type cluster despite variation in gestational time points, perhaps reflecting the fairly narrow time window of sampling.

We next examined how similar the gene expression profiles were between samples. We found that, in both adult dogs and embryos, samples from a given tissue cluster across individuals, and are distinct from samples of other tissue types (Figure 5A). Samples from similar tissue types (for example, various samples from brain or heart) also tended to cluster closer together. This is consistent with the expectation that specific tissues have distinct transcriptomes that support their specific physiological roles and that are broadly consistent across individuals [92].

Of our five adult dogs, two had strikingly similar phenotypes. Both were purebred Malinois, of similar ages (three and four years old), and were euthanized because of behavioral problems. Genetic analysis suggests relatedness equivalent to third degree relatives (12% of genome identical by descent).The other three adult dogs were over the age of ten. One dog (Adult 1) weighed just 4 kg, ~10 fold smaller than the other four adults.

**Figure 6:**
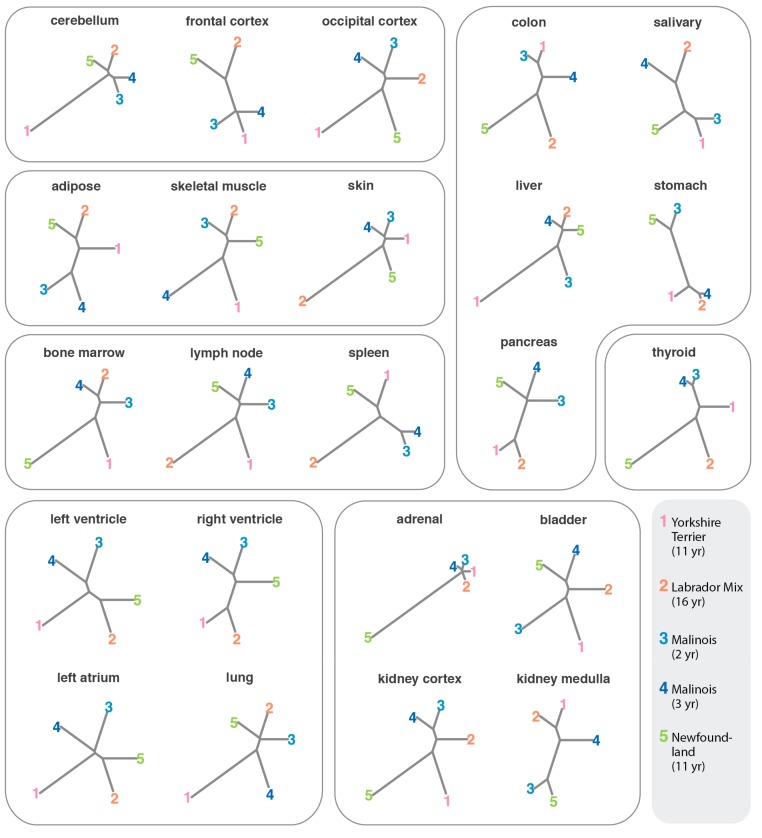
The relationship between samples within a single tissue type is highly variable. Clustering is based on Euclidean distances among samples, with no consistent clustering by age or breed observed. Outlines group tissues of a given class.

We saw no indications that the overall pattern of gene expression in tissues correlated with breed, age, or size. The two Malinois did not cluster together when the data from 23 tissue types was concatenated, and the small dog was not an outlier (Figure 5B). When tissues were examined separately, the tree topology was highly variable. The two Malinois did not appear to cluster together more frequently than other pairs of dogs, nor was the small dog an outlier (Figure 6). This is not surprising, as we are comparing gene expression across all genes, and studies in humans show both that expression levels are strongly heritable for only a small subset of genes [93], and that subtle changes in expression, or changes involving rarer cell types, may be masked in tissue-level data [94].

### 3.5. Comparison of Expression Profiles in Canine and Human Tissues

**Figure 7:**
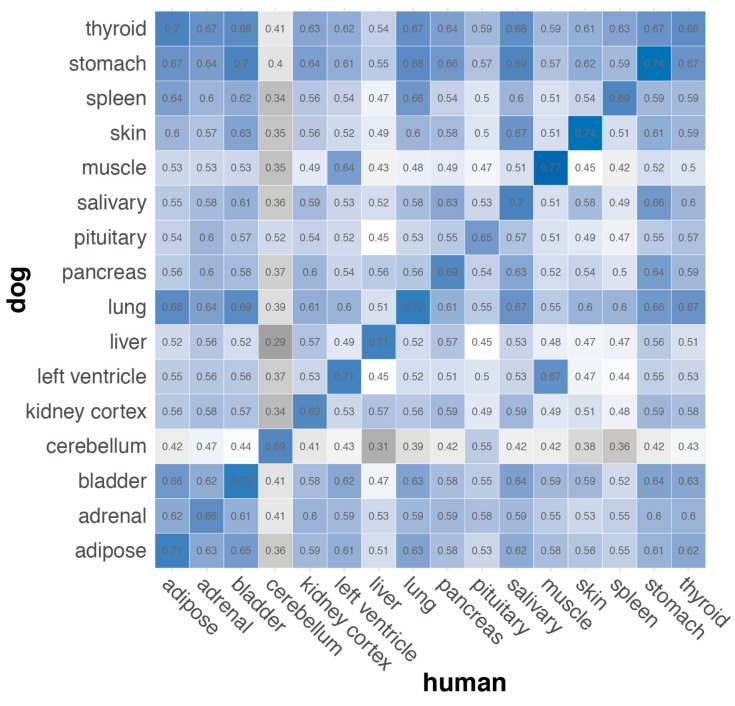
Gene expression levels correlate between dog and human tissues. Heatmap showing Spearman correlation between the genes expressed in canine and human tissues, after filtering for minimum expression (median CPM > 1) and unique orthology mapping between species. In all cases except one (dog thyroid), comparison of dog tissue to the corresponding human tissue had the highest Spearman coefficient, suggesting broad conservation of the transcriptome in these tissues across species.

Substantial congruity between human and canine tissue-specific transcriptomes is evident from a comparison of our new BarkBase RNA-seq data to human RNA-seq data publicly available through the GTEx Portal [95]. We assessed this simply by measuring the rank-order correlation of the single-gene expression values for genes expressed in dog and human tissues. While this correlation metric varies only moderately across tissues, the strongest correlation was almost always between the same tissue type in dogs and human (100% of human tissues and 94% of dog tissues; Figure 7). Human and dog skeletal muscle had the highest correlation (r^2^ = 0.77), and pituitary gland the lowest (r^2^ = 0.65). The only tissue not to match the same tissue type in the other species was dog thyroid tissue, which matched human adipose (r^2^ = 0.70) slightly better than human thyroid (r^2^ = 0.68). Comparing cerebellum with any tissue type other than cerebellum (r^2^ = 0.69) yielded some of the lowest correlations, with all except pituitary (r^2^ = 0.52–0.55) between r^2^ = 0.29 and r^2^ = 0.47. This is consistent with the tight clustering of cerebellum samples as a distinct clade on our tissue dendrogram (Figure 5A) and with previous reports that cerebellum is a complex tissue composed of a large number of cell types with transcriptomic profiles distinct from those in other organs [96].

Same tissue comparisons between dog and human yielded higher correlations (mean r^2^ = 0.71 ± 0.03) than comparing the different tissues between dog and human (mean = 0.55 ± 0.08) and than comparing different tissues within dog or human (0.62 ± 0.11). Together, these results suggest that tissue-specific dog transcriptomes have much in common with human transcriptomes of the corresponding tissues. Precisely why our data indicated a difference between the same tissue in dog and human will require further work, including determining whether the source of the deviation is primarily artifactual, arising from differences in sampling, data generation and analysis, or whether it reflects true biological differences.

### 3.6. RNA-Sequencing of Embryonic Tissues at Multiple Time Points

RNA-seq data from embryos revealed strong overlap of data for individual tissues assayed at various gestational stages as assessed by both cumulative transcription across the 1000 top-expressed genes (Figure 4), and by tissue-level clustering (Figure 5). To identify the top differentially expressed functional categories in each tissue, we first mapped all genes with FDR < 0.1 to their known human ortholog. In total, 158 genes with human orthologs were differentially expressed between embryonic data 36 and day 44 across head, heart, kidney, and liver, with the number varying by tissue type: 71 genes for head, 41 genes for heart, two genes for kidney, and 44 genes for liver. In the lung, 8365 genes were differentially expressed. These two embryonic time points were both different individuals and different sexes, contributing to differences in expression.

We analyzed these sets with an ingenuity pathway analysis and identified the top “Diseases and Bio Functions” (Table 1). The most significantly enriched set was “epithelial neoplasm" in the lung(*p* = 2 × 10^−323^), consistent with ongoing differentiation of lung epithelial cells across mammalian gestation [97]. In the head, both top sets were related to organ enlargement: hypertrophy (*p* = 9.8 × 10^−10^) and visceromegaly (*p* = 1.6 × 10^−9^). In the heart, the top set, “Morphogenesis of embryonic skeleton" (*p* = 4.4 × 10^−12^), included seven *HOXA* genes and one *HOXB* gene. *HOX* genes encode proteins whose spatial and temporal regulation guides embryonic development, and *HOXA* gene mutations are implicated in congenital heart defects [98,99]. In the kidneys, there was only one gene underlying the functional enrichment, *EMX2*, a gene involved in both urogenital and neurological development [100]. In liver, the top function was a liver-related disease (Hepatitis B virus-related hepatocellular carcinoma, *p* = 9.2 × 10^−5^). Other top sets included hyperphenylalaninemia (*p* = 2.3 × 10^−4^), a disease resulting from a lack of a liver-specific enzyme, and metabolism of acylglycerol (*p* = 3.2 × 10^−4^), a pathway involving the liver.

### 3.7. ATAC-seq

ATAC-seq (assay for transposase-accessible chromatin using sequencing) assesses chromatin accessibility genome-wide [101,102]. Pairing ATAC-seq data with RNA-seq data places the transcriptome in the context of chromatin states genomewide. This enables identification of *cis*-regulatory elements, such as enhancers, promoters, and insulators, which are not detectable using RNA-seq alone, as well as validation of novel transcription start sites relevant to a specific set of RNAs [101,103].

**Figure 8:**
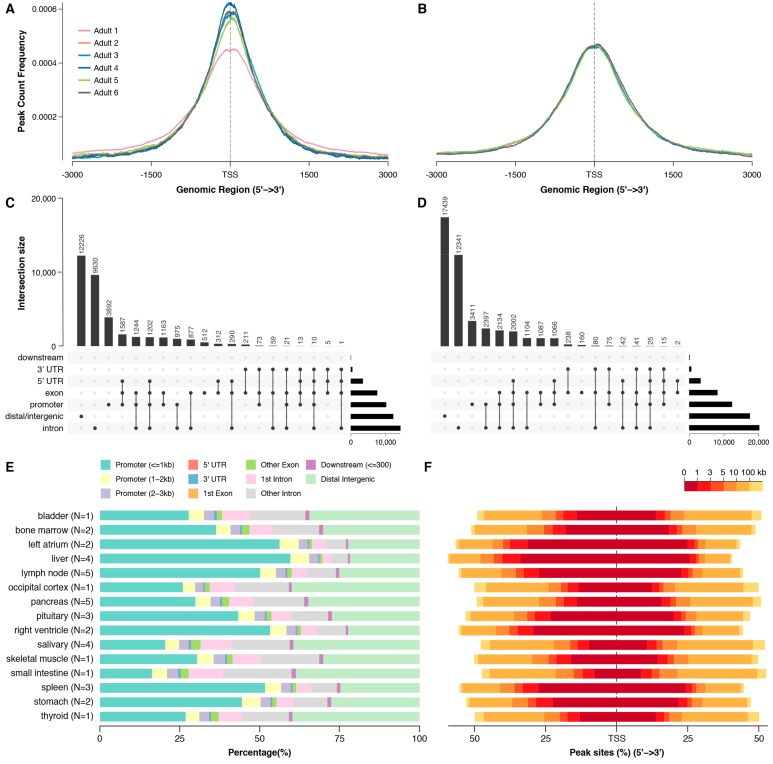
ATAC-seq maps transposase-accessible chromatin in canine tissues. Analysis of the two tissue types with ATAC-seq data for five individuals, pancreas (**A**) and salivary gland (**B**), reveals strong enrichment of peaks around known transcription start sites. This enrichment is consistent across individuals. Annotating the ATAC-seq peaks with ChIPseeker, using the Ensembl annotation of dog, shows, as expected, an overlap with known promoters in both (**C**) the pancreas (*n* ≅ 10,000) and (**D**) salivary gland (*n* ≅ 12,000), but there are more peaks in distal/intergenic regions, potentially marking novel promoters or distal regulatory elements. (**E**) Across all tissues, ATAC-seq peaks are most likely to be in annotated promoters, but a large proportion are far from genes. (**F**) In all tissues, the enrichment for ATAC-seq peaks falls off rapidly with increasing distance from a TSS.

BarkBase includes a pilot set of ATAC-seq data for 15 tissues sampled from five individual adult dogs (Figure 1). Of 46 ATAC-seq libraries created, 37 yielded sufficiently high quality data for inclusion. Each of these 37 samples has between 4600 and 136,000 ATAC-seq peaks (median 50,000). Known transcription start sites (TSS) are highly enriched for ATAC-seq peaks. A close look at two tissue types for which we have data from multiple individuals (pancreas and salivary gland) shows the pattern of enrichment is similar across individuals, but varies between tissue types (Figure 8A,B). Moreover, most of the peaks are either intronic or intergenic, illustrating the capacity of ATAC-seq to annotate potentially important genomic loci not found using RNA-seq alone. This pattern persists across all tissues tested, with 20–40% of peaks falling in distal intergenic regions and strong enrichment for transcription start sites (Figure 8C,D).

For those tissues for which we have multiple samples, we also find that large numbers of peaks are shared among tissues of the same type, as well as a significant number of peaks that are shared across all members of the same tissue, but are present in no other tissue. For example, we find 35,000 peaks shared across the five pancreas samples; of these, 8000 are unique to the pancreas. This combination suggests both that the peaks we see are reflective of real-world differences, and that we have captured regulatory elements specific to tissue type.

### 3.8. Novel Genes and ATAC

**Figure 9:**
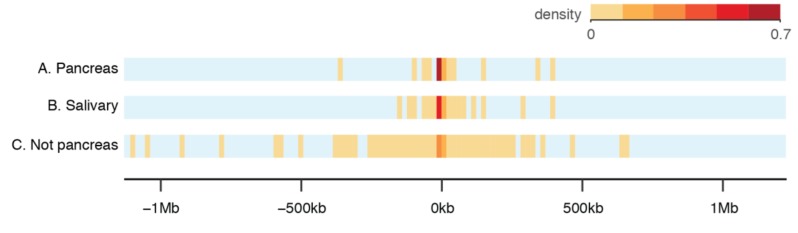
Integrating ATAC-seq with RNA-seq data can help validate novel genes. (**A**) Of the 44 novel genes expressed in the pancreas, most are less than 25 kb from a pancreas ATAC-seq peak. For those closest to ATAC-seq peaks, integrating RNA-seq and ATAC-seq provides additional evidence that they are real genes. (**B**) 58 novel genes expressed in the salivary gland (including 15 also expressed in pancreas) do not cluster as closely to pancreas ATAC-seq peaks, suggesting tissue specificity. (**C**) 491 novel genes not expressed in the pancreas are much more dispersed relative to the ATAC-seq peaks in the pancreas.

BarkBase includes 84 Mb of transcribed sequence that is not found in the existing Ensembl canine reference annotation. This includes 769 novel, multi-exon genes. We assessed the potential for ATAC-seq to discern which genes are most likely to be real, focusing on the pancreas, the tissue for which we had the most data (Figure 1). After excluding genes with a mean expression under 0.16 CPM in all samples, we found 44 novel genes expressed in the pancreas in at least two dogs, 58 novel genes expressed in the salivary gland in at least two dogs, and 491 novel genes not expressed in the pancreas in any dogs. We observed that novel genes expressed in the pancreas tended to be closer in proximity to a pancreas ATAC-seq peak than novel genes expressed in a different tissue (salivary gland), or novel genes expressed in any tissue but pancreas (Figure 9).

## 4. Discussion

BarkBase contains the largest and most comprehensive set of canine functional genomic data produced to date. Overall, data quality is high. Comparing either RNA-seq or ATAC-seq data from different individuals for a particular tissue type shows the high degree of congruence expected from previous work in humans [95,104]. RNA-seq data sets for up to 27 tissues from each of the five dogs are available for immediate download at BarkBase.org; corresponding ATAC-seq data sets discussed here are available as well. Libraries have been constructed for most of the remaining samples, and data will be posted on BarkBase as soon as sequencing is complete.

BarkBase improves substantially on earlier annotations of the dog genome. By analyzing data directly from dog tissues, rather than making inferences from human and lifting them over to the dog genome, our method improves sensitivity by enabling detection of genes expressed in dogs but not humans. With RNA-seq data from 150 samples from diverse tissues, including embryonic tissue, we detect novel genes not identified in earlier RNA-seq informed annotations. These newly identified genes include five orthologous to protein-coding genes known from human studies to function in health-relevant processes including tooth development (*STATH*) and immune function (*AHNAK2, EPX*). In addition, these five genes have been identified in various roles in cancer studies [105,106,107,108], highlighting the utility of BarkBase as a tool for advancing dog as a model for human medicine.

BarkBase is complementary to existing annotations, which include genes and transcripts that we miss. Integrating these “lost” transcripts should further improve the annotation. The genes missing from BarkBase are largely non-coding genes without human orthologs, including multi-exon lncRNAs. lncRNAs tend to have more tissue-specific expression patterns [109], including in dogs [25], and we may have missed them because of the difference in tissue types represented between our dataset and others. However, non-coding genes are also challenging to annotate. Using RNA-seq data alone, as was done for both Ensembl and BarkBase, is only the first step [110]. ATAC-seq data from BarkBase adds another level of information, guiding predictions of non-coding genes physically near the genomic elements that regulate them (Figure 9). We will integrate the ATAC-seq data into our gene predictions once the complete set of ATAC-seq data is available. Additional information will be required for the prediction of genes regulated across larger expanses of sequence [111].

Using the RNA-seq data from multiple individuals, we can compare patterns of gene expression across individuals at single-tissue resolution. We observe that samples tend to cluster by tissue type, not by individual (Figure 5A). Comparing the relationship between individuals for each tissue type, we see no evidence that particular individuals cluster together more or less often (Figure 6). This suggests that, at least within our small dataset, the effects of size, age, and breed do not substantially alter the overall transcriptional landscape. Additional data from much larger sets of individuals, with additional biological replicates and single-cell transcriptomes, may reveal subtle patterns and gene-specific differences not accessible from our present data set or analytic approach. Nonetheless, data already available through BarkBase indicate that tissue identity, not characteristics of the individual, is the strongest predictor of fundamental transcriptome features.

We note that any effort to discern breed effects on gene expression would require a much larger dataset controlled for environmental factors, such as age and lifestyle, or phenotypes, such as size (dwarfism and gigantism), that might also be expected to affect gene expression and can spuriously correlate with breed [112]. For example, in our data we observed that the shortest distance between any two organ-specific samples is between thyroid samples from Adult 3 and Adult 4, the two younger Malinois dogs. It is tempting to speculate that this is associated with the Malinois’ reported higher risk of thyroid disease, or with age-related changes in thyroid function, but we have no ability to distinguish age effects from breed effects [113,114]. Work investigating how germline variants affect gene expression in humans suggests sample sizes in the hundreds may be required [115].

We also saw very little difference in overall gene expression patterns between embryonic time points, with samples clustering almost perfectly by tissue type (Figure 5C). The two exceptions were the two head samples that had pooled multiple individuals, likely reflecting the increased variability in gene expression in these samples. This was, at first, unexpected, as the transcriptome of individual embryonic tissues is known to shift substantially across embryonic development [116]. However, we also observed that the cumulative distribution of transcripts closely matched the adult tissues (Figure 4C). One possible explanation is that our sampling of embryos more than halfway through gestation, coupled with their spread across a fairly narrow, nine-day time window, does not capture the dramatic epigenomic and transcriptomic shifts that characterize preimplantation and early developmental shifts in, for example, mouse [116,117] and human [118]. Instead, we confirm that tissue-specific embryonic transcriptomes are broadly stable over a ~10d window midway through gestation and embryonic transcriptomes, and are broadly similar to transcriptomes of matched adult tissues.

Higher resolution comparisons through differential gene expression (DGE) revealed developmental changes not evident from overall gene expression patterns. For example, IPA analysis of genes differing in expression between heart tissue sampled at these two gestational time points revealed enrichment for genes associated with abnormalities of heart development. DGE in liver revealed enrichment of genes associated with metabolism, and in lung revealed genes associated with epithelial neoplasm and cancers. These findings indicate that BarkBase will be a powerful tool for identifying specific genes and classes of genes whose activity shifts during gestation, and suggest future efforts should focus on expanding on this work to capture the full arc of transcriptomic changes across canine development in utero.

## 5. Conclusions

BarkBase dramatically expands genomic resources for dogs, improving the annotation of the canine genome and revealing close similarities between dog transcriptomes and tissue-matched data from humans. BarkBase samples already span the typical canine lifespan, including development in utero*,* and includes individuals from several different breeds, but a more comprehensive data set will be required for inference of potential associations of transcriptomic features to dog age, breed, and/or environmental factors. We therefore offer BarkBase both as a powerful resource that is available to all researchers for immediate use, and as a paradigm for ongoing collection of data to further enhance the value of dog as a powerful natural model for human disease.

## Figures and Tables

**Table 1:** Functional enrichment among genes differentially expressed in embryonic tissues at d36 and d44 reflects organ-specific roles. Genes differentially expressed in each of the five individual tissues sampled at embryonic d36 as compared d44 (FDR < 0.1) were analyzed for functional enrichment using IPA.

	Category	Diseases or Functions	*p*	No. of Genes	Genes
**head**	Organismal Injury and Abnormalities	Hypertrophy	9.8 × 10^−10^	15	*TRIM55, CXCL12, TNNI3K, TNNT2, ADGRG1, GATA6, JPH2, INHBA, RPS6KA2, NR3C1, SLC25A4, CAST, RRAD, TRIM63, IL33*
		Visceromegaly	1.6 × 10^−9^	18	*TRIM55, CXCL12, TNNI3K, TNNT2, NEXN, ADGRG1, GATA6, JPH2, INHBA, TBX20, NR3C1, SLC25A4, CAST, RRAD, SSTR2, BIK, TRIM63, IL33*
	Cardiovascular Disease, Cardiovascular System Development and Function, Organ Morphology, Organismal Development, Organismal Injury and Abnormalities	Enlargement of heart	3.7 × 10^−9^	15	*TRIM55, CXCL12, TNNI3K, TNNT2, NEXN, ADGRG1, GATA6, JPH2, INHBA, TBX20, SLC25A4, CAST, RRAD, TRIM63, IL33*
		Abnormal morphology of heart	5.0 × 10^−9^	17	*TRIM55, CXCL12, TNNI3K, TNNT2, NEXN, ADGRG1, GATA6, JPH2, DHRS3, INHBA, TBX20, RPS6KA2, SLC25A4, CAST, RRAD, TRIM63, IL33*
		Muscular hypertrophy	5.7 × 10^−9^	10	*INHBA, TRIM55, RPS6KA2, CAST, RRAD, ADGRG1, GATA6, JPH2, TRIM63, IL33*
		Hypertrophy of heart	1.6 × 10^−7^	11	*CXCL12, INHBA, TRIM55, TNNI3K, SLC25A4, TNNT2, RRAD, ADGRG1, GATA6, TRIM63, IL33*
	Cardiovascular System Development and Function	Morphology of cardiovascular system	6.4 × 10^−9^	19	*CXCL12, TRIM55, TNNI3K, TNNT2, PLA2G7, NEXN, ADGRG1, GATA6, JPH2, DHRS3, INHBA, TBX20, RPS6KA2, SLC25A4, RRAD, CAST, SSTR2, TRIM63, IL33*
	Cardiovascular Disease, Cardiovascular System Development and Function	Abnormal morphology of cardiovascular system	8.2 × 10^−9^	18	*TRIM55, CXCL12, TNNI3K, TNNT2, NEXN, ADGRG1, GATA6, JPH2, DHRS3, INHBA, TBX20, RPS6KA2, SLC25A4, CAST, RRAD, SSTR2, TRIM63, IL33*
	Organismal Development, Organismal Injury and Abnormalities	Abnormal morphology of thoracic cavity	2.9 × 10^−8^	18	*TRIM55, CXCL12, TNNI3K, TNNT2, NEXN, ADGRG1, GATA6, JPH2, DHRS3, INHBA, TBX20, RPS6KA2, NR3C1, SLC25A4, CAST, RRAD, TRIM63, IL33*
	Organismal Development	Abnormal morphology of body cavity	8.0 × 10^−8^	22	*TRIM55, RBMS1, CXCL12, TNNI3K, TNNT2, MAPK8IP2, NEXN, ADGRG1, GATA6, JPH2, DHRS3, INHBA, TBX20, RPS6KA2, NR3C1, SLC25A4, RRAD, CAST, BIK, SSTR2, TRIM63, IL33*
**heart**	Skeletal and Muscular System Development and Function	Morphogenesis of embryonic skeleton	4.4 × 10^−12^	7	*HOXB8, HOXA6, HOXA3, HOXA7, HOXA4, HOXA2, HOXA5*
		Morphology of axial skeleton	1.1 × 10^−8^	8	*HSD11B2, HOXB8, HOXA3, HOXA6, HOXB9, HOXA4, HOXA5, mir-196*
		Fusion of bone	1.6 × 10^−8^	6	*HOXA6, HOXA3, HOXB9, HOXA7, HOXA4, HOXA5*
		Morphology of skeleton	1.8 × 10^−8^	9	*HSD11B2, HOXB8, HOXA6, HOXA3, HOXB9, HOXA4, HOXA2, mir-196, HOXA5*
	Embryonic Development, Organismal Development	Patterning of rostrocaudal axis	1.2 × 10^−11^	8	*HOXB8, HOXA6, HOXA3, HOXB9, HOXA7, HOXA4, HOXA2, HOXA5*
	Organismal Development	Abnormal morphology of body cavity	9.2 × 10^−9^	17	*TRIM55, HSD11B2, MYH7, SMYD1, TNNC1, TNNI3K, HOXA3, HOXB9, ATP2A2, HOXA5, PDZK1, TBX20, SLC25A4, HOXA7, HOXA2, TRIM63, SGPP2*
	Cardiovascular System Development and Function, Organ Development, Organ Morphology, Skeletal and Muscular System Development and Function	Contraction of cardiac muscle	3.9 × 10^−8^	6	*MYH7, TNNC1, TNNI3K, ATP2A2, TRIM63, SRL*
	Organ Morphology, Skeletal and Muscular System Development and Function	Quantity of rib	6.0 × 10^−8^	5	*HOXA6, HOXB9, HOXA4, HOXA5, mir-196*
	Cancer, Skeletal and Muscular Disorders, Tissue Morphology	Transformation of vertebrae	7.1 × 10^−8^	5	*HOXA6, HOXB9, HOXA4, HOXA5, mir-196*
	Organismal Development, Organismal Injury and Abnormalities	Abnormal morphology of thoracic cavity	8.8 × 10^−8^	13	*TRIM55, MYH7, SMYD1, TNNC1, TNNI3K, HOXA3, HOXB9, ATP2A2, HOXA5, TBX20, SLC25A4, HOXA7, TRIM63*
**kidney**	Cell Cycle	Cell division of neural stem cells	9.1 × 10^−5^	1	*EMX2*
	Embryonic Development, Nervous System Development and Function, Organ Development, Organismal Development, Tissue Development	Development of hippocampal fissure	9.1 × 10^−5^	1	*EMX2*
	Nervous System Development and Function, Organ Morphology, Organismal Development	Size of primary visual cortex	9.1 × 10^−5^	1	*EMX2*
	Nervous System Development and Function, Neurological Disease, Organ Morphology, Organismal Development, Organismal Injury and Abnormalities	Abnormal morphology of medial ganglionic eminences	1.8 × 10^−4^	1	*EMX2*
	Developmental Disorder, Embryonic Development, Tissue Morphology	Degeneration of Wolffian duct	1.8 × 10^−4^	1	*EMX2*
**liver**	Cancer, Gastrointestinal Disease, Hepatic System Disease, Organismal Injury and Abnormalities	Hepatitis B virus-related hepatocellular carcinoma	9.2 × 10^−5^	3	*TF, ALDOB, RGN*
	Cell-To-Cell Signaling and Interaction, Renal and Urological System Development and Function	Activation of kidney cells	9.8 × 10^−5^	2	*TF, MST1*
	Organismal Injury and Abnormalities	Organ Degeneration	9.8 × 10^−5^	8	*EFEMP1, GSTZ1, TF, GRID2, RP2, RGN, ZNF408, mir-22*
	Developmental Disorder, Hereditary Disorder, Metabolic Disease, Organismal Injury and Abnormalities	Hyperphenylalaninemia	2.3 × 10^−4^	2	*GCH1, DNAJC12*
	Lipid Metabolism, Small Molecule Biochemistry	Metabolism of acylglycerol	3.2 × 10^−4^	4	*ACSL5, SLC22A4, RGN, F2*
lung	Cancer,Organismal Injury and Abnormalities	Epithelial neoplasm	0.0	6961	*many*
		Non-hematological solid tumor	0.0	7039	*many*
		Nonhematologic malignant neoplasm	0.0	7021	*many*
		Carcinoma	0.0	6949	*many*
		Tumorigenesis of tissue	0.0	6969	*many*

## Supplementary Materials

The following are available online at https://www.mdpi.com/2073-4425/10/6/433/s1: Table S1. All samples in BarkBase. Table S2. Variant Calling Background Information. Table S3. Sensitivity to detect annotated exons. Table S4. Ensembl genes detected in BarkBase. Table S5. Ensembl genes not seen in BarkBase. Table S6. GO enrichment of Ensembl genes not seen in BarkBase. Table S7. GO enrichment of Ensembl genes detected in BarkBase. Table S8. Human genes overlapped by novel BarkBase transcripts.
