# Supplementary material for: BarkBase: Epigenomic Annotation of Canine Genomes
Source: Genes (Basel). 2019 Jun 7;10(6):433. doi: 10.3390/genes10060433 (PMC6627511; doi:10.3390/genes10060433)
Supplement: Supplementary file 1 [file genes-10-00433-s001.zip › SI/TableS3.pdf]

Table S3. Sensitivity at exon level

The sensitivity of each of the three datasets to detect exons in the other two datasets was determined by calculating sensitivity using the GffCompare tool for each dataset using the other datasets as reference in turn. Sensitivity was calculated as the total number of exons in the reference annotation identified by the query dataset, divided by the total number of exons in the reference dataset [40]. Both the BarkBase and Hoeppner et al datasets had high sensitivity to detect exons in the Ensembl dataset. The Ensembl and Hoeppner et al datasets each had an approximately 50% sensitivity to detect exons in BarkBase, suggesting that BarkBase contains novel exons not discovered in the other datasets

		**Reference**		
		Ensembl	BarkBase	Hoeppner
**Query**	Ensembl		47.1%	51.7%
	BarkBase	87.6%		56.6%
	Hoeppner	90.5%	55.2%	
