# Supplementary material for: BarkBase: Epigenomic Annotation of Canine Genomes
Source: Genes (Basel). 2019 Jun 7;10(6):433. doi: 10.3390/genes10060433 (PMC6627511; doi:10.3390/genes10060433)
Supplement: Supplementary file 1 [file genes-10-00433-s001.zip › SI/TableS8.pdf]

Table S8: Human genes overlapped by novel BarkBase transcripts. Fifteen transcripts in the BarkBase RNA-seq data which did not overlap any annotation in Ensembl or Hoeppner *et al* overlapped five human genes in the UCSC Other RefSeq track.

**RefSeq accession**	**Gene name**	**Gene description**	**Annotation**
NM_000502	*EPX*	eosinophil peroxidase	No
NM_000523	*HOXD13*	homeobox D13	No
NM_003154	*STATH*	statherin	No
NM_001160264	*FEZF1*	FEZ family zinc finger 1	No
NM_001350929	*AHNAK2*	AHNAK nucleoprotein 2	Yes, non-unique alignment, additional exons
